# Flexible transparent displays based on core/shell upconversion nanophosphor-incorporated polymer waveguides

**DOI:** 10.1038/srep45659

**Published:** 2017-04-03

**Authors:** Bong Je Park, A-Ra Hong, Suntak Park, Ki-Uk Kyung, Kwangyeol Lee, Ho Seong Jang

**Affiliations:** 1 Electronics and Telecommunications Research Institute (ETRI), 218 Gajeong-ro, Yuseong-gu, Daejeon 34129, Republic of Korea; 2 Materials Architecturing Research Center, Korea Institute of Science and Technology, 5, Hwarang-ro 14-gil, Seongbuk-gu, Seoul 02792, Republic of Korea; 3 Department of Chemistry, Korea University, 145 Anam-ro, Seongbuk-gu, Seoul 02841, Republic of Korea; 4 Department of Nanomaterials Science and Engineering, Korea University of Science and Technology, 218 Gajeong-ro, Yuseong-gu, Daejeon 34113, Republic of Korea

## Abstract

Core/shell (C/S)-structured upconversion nanophosphor (UCNP)-incorporated polymer waveguide-based flexible transparent displays are demonstrated. Bright green- and blue-emitting Li(Gd,Y)F_4_:Yb,Er and Li(Gd,Y)F_4_:Yb,Tm UCNPs are synthesized via solution chemical route. Their upconversion luminescence (UCL) intensities are enhanced by the formation of C/S structure with LiYF_4_ shell. The Li(Gd,Y)F_4_:Yb,Er/LiYF_4_ and Li(Gd,Y)F_4_:Yb,Tm/LiYF_4_ C/S UCNPs exhibit 3.3 and 2.0 times higher UCL intensities than core counterparts, respectively. In addition, NaGdF_4_:Yb,Tm/NaGdF_4_:Eu C/S UCNPs are synthesized and they show red emission via energy transfer and migration of Yb^3+^ → Tm^3+^ → Gd^3+^ → Eu^3+^. The C/S UCNPs are incorporated into bisphenol A ethoxylate diacrylate which is used as a core material of polymer waveguides. The fabricated stripe-type polymer waveguides are highly flexible and transparent (transmittance > 90% in spectral range of 443–900 nm). The polymer waveguides exhibit bright blue, green, and red luminescence, depending on the incorporated UCNPs into the polymer core, under coupling with a near infrared (NIR) laser. Moreover, patterned polymer waveguide-based display devices are fabricated by reactive ion etching process and they realize bright blue-, green-, and red-colored characters under coupling with an NIR laser.

Recently, upconversion nanophosphors (UCNPs) have attracted great attention due to unique optical properties such as anti-Stokes shift luminescence unlike conventional luminescent materials showing downconversion luminescence (including downshifting and quantum splitting)^1^,^2^,^3^. The advancement of solution chemical synthesis of the UCNPs boosted the explosive researches on the UCNPs’ syntheses and applications^4^,^5^,^6^,^7^,^8^,^9^. Although quantum yields of the UCNPs are low compared with downconversion materials^5^, they have potential applications to biological field. Near infrared (NIR) light which is used as an excitation source for the UCNPs can penetrate deeply into biological system compared with ultraviolet (UV) or visible light, and it causes much smaller damage to biomolecules as well^10^,^11^,^12^. Moreover, clear fluorescence image with high signal to noise ratio can be obtained by using the UCNPs and NIR light source because the NIR light does not induce autofluorescence from the biomolecules^12^,^13^. Although many studies on bio-imaging using the UCNPs have been reported thanks to the aforementioned advantages utilizing the UCNPs and NIR light source^14^,^15^,^16^,^17^, the applications of the UCNPs are not restricted to bio-related field.

The UCNPs can also be applied to new emissive displays such as transparent three dimensional (3D) volumetric displays^8^,^18^,^19^,^20^. With the development of efficient 980 nm diode lasers, the probability that the upconversion (UC) materials are used in practical applications becomes high^21^. Since trace of excitation light sources should not be visible in the transparent 3D displays, the UCNPs, which are excited with invisible NIR light, can be a promising material for realizing transparent 3D displays. However, because the 3D volumetric display devices exhibit 3D images in real 3D spatial form^18^, the realization of 3D volumetric displays indispensably requires a thicker nature of the display devices. On the other hand, when the thickness of the transparent display devices is thin enough to be bendable, transparent and flexible display devices using the UCNPs can be actualized. Polymer waveguides have a capability of application to flexible displays and Park *et al*. recently reported polymer waveguide-based thin film displays^22^,^23^. These results encourage us to develop transparent and flexible display devices by combining the UCNPs with polymer waveguide. We previously reported bright green-emitting Li(Gd,Y)F_4_:Yb,Er UCNPs which have high color purity of 98.9%^24^. Small-sized Li(Gd,Y)F_4_:Yb,Er UCNPs can be readily synthesized by adjusting the ratio of Gd to Y, and it can reduce light scattering for the waveguide containing the UCNPs because light scattering coefficient increases with particle size^25^. Whereas smaller size of the UCNPs decreases light scattering, leading to higher transparency of the devices, it causes PL intensity of the UCNPs to decrease^26^,^27^. The issue of weaker PL intensity of smaller UCNPs is mainly attributed to large surface defect density and it can be overcome by suppressing the surface quenching effect via formation of shell on the UCNP core^3^,^26^,^27^,^28^,^29^. In this study, Li(Gd,Y)F_4_:Yb,Er/LiYF_4_ core/shell (C/S) UCNPs were synthesized to enhance green UC luminescence (UCL) and applied to the polymer waveguide-based flexible transparent displays. Recently, arrayed waveguide gratings on green-emitting UCNP layer by micromolding in capillaries were reported^30^. In the transparent film, patterned discrete UCNP agglomerates were placed between arrayed waveguide gratings surrounded by polydimethyl siloxane polymer with millimeter scale thickness and it shows that UCNPs can be applied to waveguides^30^. In this study, C/S-structured UCNPs were incorporated into a polymer, and highly thin and bright flexible transparent displays were fabricated using UCNP-incorporated polymer waveguides. To fabricate blue-emitting flexible transparent displays, Li(Gd,Y)F_4_:Yb,Tm/LiYF_4_ C/S UCNPs were synthesized because Tm^3+^ is known as one of the efficient blue-emitting activators^31^,^32^. Additionally, for red-emitting UCNPs, we synthesized NaGdF_4_:Yb,Tm/NaGdF_4_:Eu C/S UCNPs by adopting the method previously reported by Wang *et al*.^33^ because Eu^3+^ emits strong red emission peak via energy migration process under excitation with NIR light^33^. These green-, blue-, and red-emitting C/S UCNPs were incorporated into the polymer waveguides and the UCNP-incorporated polymer waveguide-based bright flexible transparent displays with highly thin thickness are demonstrated here for the first time.

## Results and Discussion

### Morphologies of UCNPs

**Figure 1:**
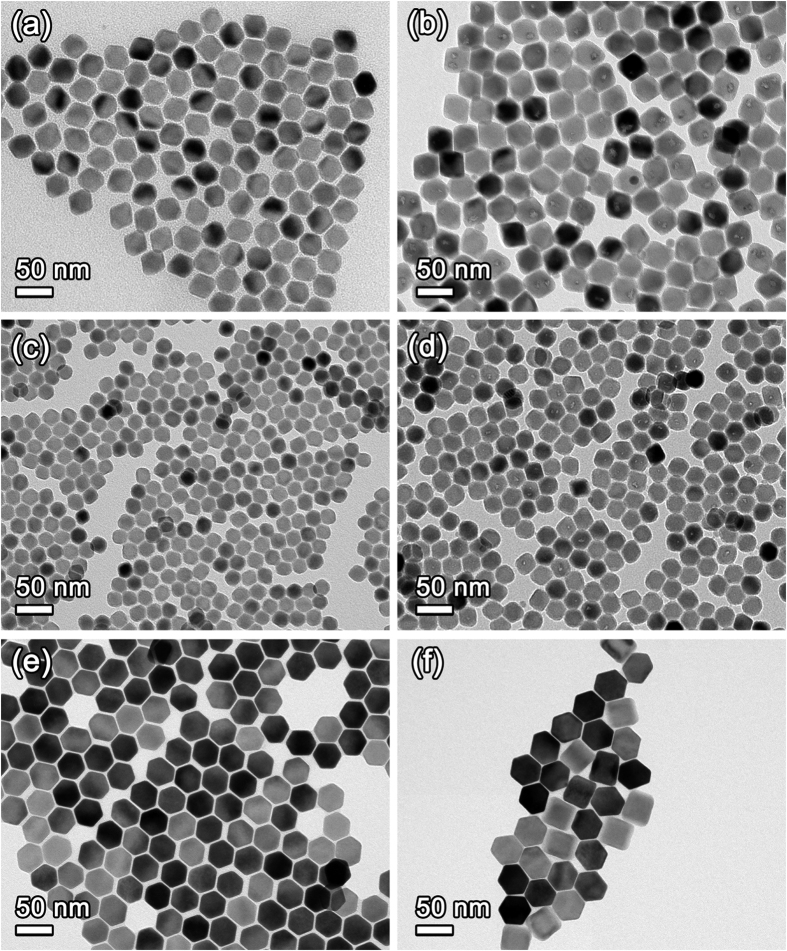
TEM images of (**a**) Li(Gd,Y)F_4_:Yb,Er, (**b**) Li(Gd,Y)F_4_:Yb,Er/LiYF_4_, (**c**) Li(Gd,Y)F_4_:Yb,Tm, (**d**) Li(Gd,Y)F_4_:Yb,Tm/LiYF_4_, (**e**) NaGdF_4_:Yb,Tm, and (**f**) NaGdF_4_:Yb,Tm/NaGdF_4_:Eu UCNPs.

Because the shell formation on the core UCNPs significantly enhances UCL as mentioned above^34^,^35^, C/S structure is believed to be crucial for UCNPs’ practical applications. Thus, there have been many studies on the C/S-structured UCNPs and, in particular, C/S UCNPs based on NaREF_4_:Yb,Er(Tm) where RE represents rare earth elements such as Y, Gd, and Lu etc. were mainly reported^3^. It was also reported that LiREF_4_-based C/S UCNPs exhibit bright luminescence^31^,^32^,^36^. In the case of LiGdF_4_, there are some reports on the difficulty of the synthesis of LiGdF_4_ nanocrystals with tetragonal structure via solution chemical route^24^,^37^. Thus, we modified material composition by substituting Gd with Y for the formation of single tetragonal-phased nanocrystals^24^. In this study, we report bright green- and blue-emitting LiYF_4_-coated Li(Gd,Y)F_4_:Yb,Er(Tm) C/S UCNPs with tetragonal structure, inducing tetragonal bipyramidal shape^38^. The transmission electron microscopy (TEM) images of the core and C/S UCNPs are shown in Fig. 1. Truncated parallelogram or hexagonal shapes are observed in the TEM images of Li(Gd,Y)F_4_:Yb,Er and Li(Gd,Y)F_4_:Yb,Tm core UCNPs. In the TEM images of Fig. 1a–d, truncated parallelogram shape is mostly observed and the shape is attributed to 2D projection of the tetragonal bipyramidal morphology of the synthesized Li(Gd,Y)F_4_:Yb,Er and Li(Gd,Y)F_4_:Yb,Tm UCNPs with blunt tips^38^. The synthesized Li(Gd,Y)F_4_:Yb,Er and Li(Gd,Y)F_4_:Yb,Tm cores, and Li(Gd,Y)F_4_:Yb,Er/LiYF_4_ and Li(Gd,Y)F_4_:Yb,Tm/LiYF_4_ C/S UCNPs have tetragonal structure judging from the measured spacing of 0.47 nm between two adjacent lattice fringes, which are in good agreement with *d*-spacing between {101} planes (*d*_101_ = 0.465 nm) of LiYF_4_ with tetragonal structure (Figures S1–S4). The high-resolution TEM (HR-TEM) and high angle annular dark field (HAADF) high-resolution scanning transmission electron microscopy (HR-STEM) images indicate that the synthesized Li(Gd,Y)F_4_:Yb,Er and Li(Gd,Y)F_4_:Yb,Tm cores and Li(Gd,Y)F_4_:Yb,Er/LiYF_4_ and Li(Gd,Y)F_4_:Yb,Tm/LiYF_4_ C/S UCNPs are single crystalline, as shown in Figures S1–S4. In the HR-TEM and HR-STEM images of C/S UCNPs, lattice mismatch between core and shell was not observed, indicating that the LiYF_4_ shells were epitaxially grown on the Li(Gd,Y)F_4_:Yb,Er and Li(Gd,Y)F_4_:Yb,Tm cores. The epitaxial growth of LiYF_4_ shell on the core is due to the same crystal structure of core composition and shell composition with similar lattice parameter^39^. On the other hand, highly ordered hexagonal-shaped particles are viewed in the TEM image of the NaGdF_4_:Yb,Tm core UCNPs and the observed shape is attributed to the alignment of NaGdF_4_:Yb,Tm UCNPs with hexagonal prism morphology along < 0001 > direction (Fig. 1e). The rectangular shape observed in TEM image of Fig. 1f is ascribed to the alignment of NaGdF_4_:Yb,Tm/NaGdF_4_:Eu C/S UCNPs along < 10
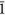
0 > direction. As shown in HR-TEM and HR-STEM images of Figures S5 and S6, NaGdF_4_-based core and C/S UCNPs have single crystalline nature with high crystallinity based on highly clear lattice fringes. The measured lattice spacings between two lattice fringes of NaGdF_4_:Yb,Tm-based core and C/S UCNPs were in agreement with *d*-spacing between (10
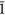
0) planes (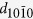 = 0.521 nm) of NaGdF_4_ with hexagonal structure. In addition, crystal structures of the core and C/S UCNPs were characterized by using X-ray diffraction (XRD) patterns. The XRD patterns shown in Figure S7 support that Li(Gd,Y)F_4_:Yb,Er- and Li(Gd,Y)F_4_:Yb,Tm-based core and C/S UCNPs have tetragonal structure and NaGdF_4_:Yb,Tm-based core and C/S UCNPs have hexagonal structure.

**Figure 2:**
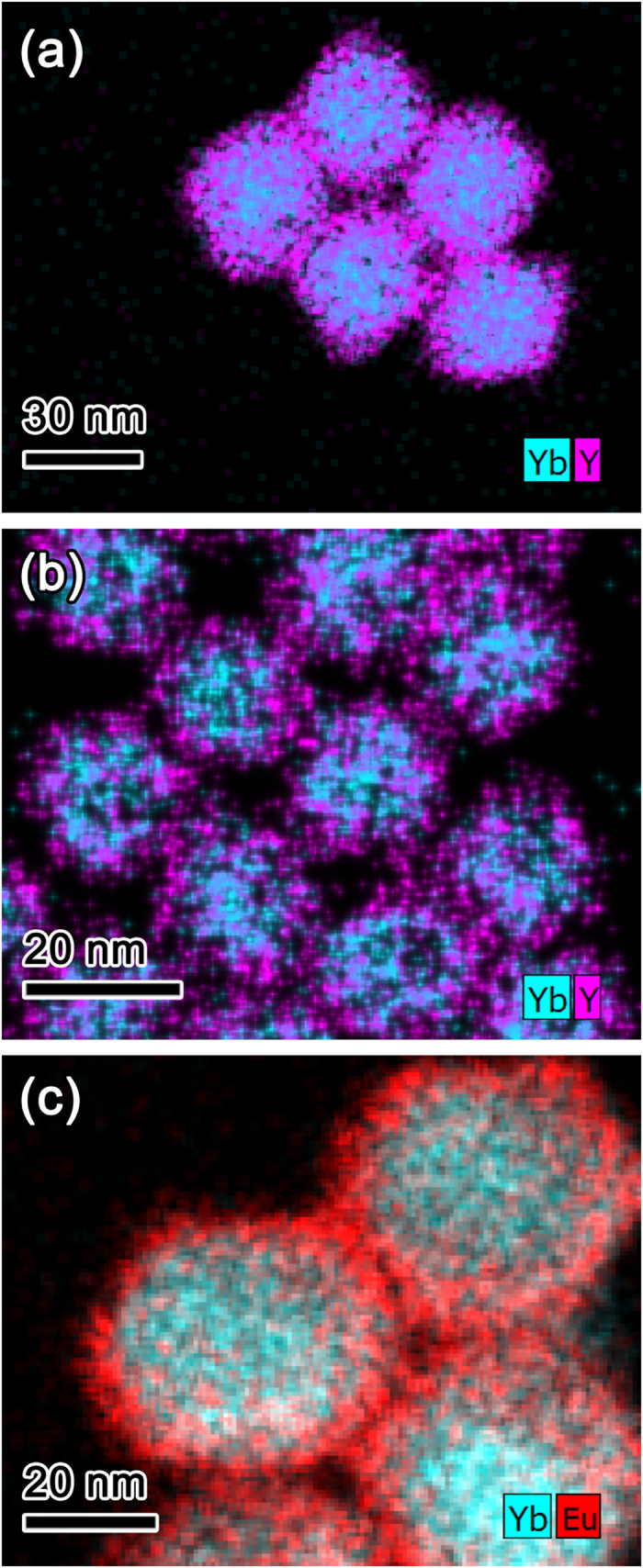
EDS maps superposed with Yb *Lα* (cyan) and Y *Kα* (magenta) for (**a**) Li(Gd,Y)F_4_:Yb,Er/LiYF_4_ UCNPs, (**b**) Li(Gd,Y)F_4_:Yb,Tm/LiYF_4_ UCNPs, and (**c**) EDS map superposed with Yb *Lα* (cyan) and Eu *Lα* (red) for NaGdF_4_:Yb,Tm/NaGdF_4_:Eu UCNPs.

After the formation of LiYF_4_ shells on the Li(Gd,Y)F_4_:Yb,Er and Li(Gd,Y)F_4_:Yb,Tm core UCNPs, the sizes of the UCNPs increased from 32.6 nm × 36.5 nm (short edge × long edge) to 36.8 nm × 41.5 nm and from 19.6 nm × 22.8 nm to 24.7 nm × 28.4 nm, respectively. Similarly, NaGdF_4_:Yb,Tm/NaGdF_4_:Eu C/S UCNPs showed larger particle size (39.5 nm) than the NaGdF_4_:Yb,Tm core UCNPs (34.6 nm) due to the growth of the NaGdF_4_:Eu layer on the NaGdF_4_:Yb,Tm core. The increase of particle size means that the shell was successfully formed on the core UCNP. Furthermore, the formation of the shell on the Li(Gd,Y)F_4_:Yb,Er, Li(Gd,Y)F_4_:Yb,Tm, and NaGdF_4_:Yb,Tm UCNP cores was directly confirmed by energy dispersive X-ray spectroscopy (EDS) analysis. The EDS maps shown in Fig. 2 apparently indicate that the C/S-structured UCNPs were successfully synthesized. It is noteworthy that the core element, Yb is located in the core region and shell elements (Y for the LiYF_4_ shell and Eu for the NaGdF_4_:Eu shell) spread out into wider region than core region, indicating successful growth of the shell on the core UCNP (Figures S8–S10).

### Luminescence properties of UCNPs

**Figure 3:**
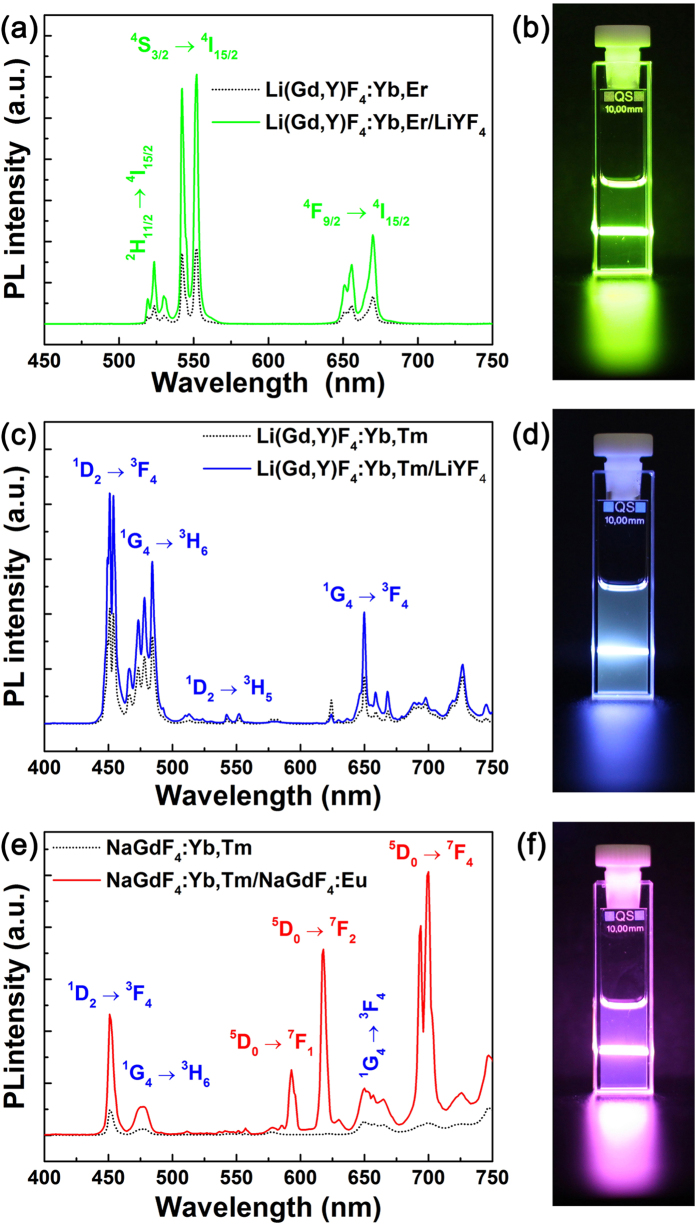
PL spectra of (**a**) Li(Gd,Y)F_4_:Yb,Er (dotted black line) and Li(Gd,Y)F_4_:Yb,Er/LiYF_4_ (solid green line), (**c**) Li(Gd,Y)F_4_:Yb,Tm (dotted black line) and Li(Gd,Y)F_4_:Yb,Tm/LiYF_4_ (solid blue line), and (**e**) NaGdF_4_:Yb,Tm (dotted black line) and NaGdF_4_:Yb,Tm/NaGdF_4_:Eu (solid red line). Photographs showing luminescence from (**b**) Li(Gd,Y)F_4_:Yb,Er/LiYF_4_, (**d**) Li(Gd,Y)F_4_:Yb,Tm/LiYF_4_, and (**f**) NaGdF_4_:Yb,Tm/NaGdF_4_:Eu C/S UCNP solutions under excitation with 980 nm NIR light.

Figure 3 shows UC photoluminescence (PL) spectra of green-, blue-, and red-emitting core and C/S UCNPs under excitation with 980 nm NIR light. In all cases, C/S UCNPs exhibited much stronger UC PL intensity than core UCNPs. In Fig. 3a, characteristic emission peaks of Er^3+^ ions are observed due to ^2^H_11/2_ and ^4^S_3/2_ → ^4^I_15/2_ transitions in green spectral region and ^4^F_9/2_ → ^4^I_15/2_ transition in red spectral region^24^. By the formation of LiYF_4_ shell, the Li(Gd,Y)F_4_:Yb,Er/LiYF_4_ C/S UCNPs exhibited 3.3 times higher PL intensity than Li(Gd,Y)F_4_:Yb,Er core UCNPs. Although emission peaks are shown in both green and red spectral region, the Li(Gd,Y)F_4_:Yb,Er/LiYF_4_ UCNPs exhibit green light due to intense emission peak attributed to ^4^S_3/2_ → ^4^I_15/2_ transition, as shown in Fig. 3b. The Li(Gd,Y)F_4_:Yb,Tm/LiYF_4_ C/S UCNPs exhibited 2.0 times higher PL intensity than Li(Gd,Y)F_4_:Yb,Tm core UCNPs and showed bright blue light via electronic transitions from ^1^D_2_ to ^3^F_4_ and from ^1^G_4_ to ^3^H_6_ in Tm^3+^ ions, as shown in Fig. 3c and d^31^,^40^. When optically inert LiYF_4_ shell was grown on the green- and blue-emitting core UCNPs, PL intensities of green and blue emissions were significantly enhanced without change of the spectral shape by suppressing surface quenching due to the decrease of surface defects such as dangling bonds^26^. However, when NaGdF_4_:Eu was coated on the NaGdF_4_:Yb,Tm core, red emission peaks were created from the Eu^3+^ ions via energy migration process through a network of the Gd sublattice in addition to PL enhancement of Tm^3+^^33^,^41^. For UC red emission, excited energy is transferred from ^1^I_6_ level of Tm^3+^ to ^6^P_7/2_ level of Gd^3+^ through five-step UC process via efficient energy transfer of Yb^3+^  → Tm^3+^ followed by energy transfer from Gd^3+^ to Eu^3+^ 33. Finally, sharp emission peaks at red spectral region are generated via strong electronic transitions of ^5^D_0_ → ^7^F_J_ (J = 1, 2, and 4) in Eu^3+^ ions^42^,^43^. As a result, the NaGdF_4_:Yb,Tm/NaGdF_4_:Eu C/S UCNPs emit reddish purple light because blue emission peaks due to ^1^D_2_ → ^3^F_4_ and ^1^G_4_ → ^3^H_6_ transitions of Tm^3+^ exist together with Eu^3+^ emission peaks, as shown in Fig. 3e and f. Although the NaGdF_4_:Yb,Tm/NaGdF_4_:Eu UCNPs do not emit pure red light, they were adopted as a red-emitting UC material due to its strong reddish UC emission under excitation with 980 nm NIR light. In our experimental condition, NaGdF_4_:Yb,Tm/NaGdF_4_:Eu UCNPs showed stronger red light than other red-emitting UCNPs such as NaGdF_4_:Yb,Ho,Ce/NaYF_4_ (Figure S11). In addition, when we compared luminescence intensity of Li(Gd,Y)F_4_:Yb,Er/LiYF_4_ with that of NaGdF_4_:Yb,Tm/NaGdF_4_:Eu, the former exhibited slightly higher PL intensity than the latter (Figure S12). When we consider UC luminescence mechanism of both UCNPs, the Li(Gd,Y)F_4_:Yb,Er/LiYF_4_ UCNPs emit green light via two-step UC process, whereas the NaGdF_4_:Yb,Tm/NaGdF_4_:Eu UCNPs exhibit blue emission from Tm^3+^ via three-step UC process and red emission from Eu^3+^ via five-step UC process, as shown in Figure S13. Therefore, it is expected that Li(Gd,Y)F_4_:Yb,Er/LiYF_4_ UCNPs show much higher PL intensity than the NaGdF_4_:Yb,Tm/NaGdF_4_:Eu UCNPs under the same excitation condition. However, the difference of PL intensity between the Li(Gd,Y)F_4_:Yb,Er/LiYF_4_ and the NaGdF_4_:Yb,Tm/NaGdF_4_:Eu UCNPs was not large as shown in Figure S12. This result is attributed to the fact that the size of NaGdF_4_:Yb,Tm/NaGdF_4_:Eu UCNPs is larger than that of the Li(Gd,Y)F_4_:Yb,Er/LiYF_4_ UCNP, because larger UCNPs exhibit stronger luminescence intensity than smaller UCNPs^26^. The related green-, blue-, and red-emitting UCL mechanisms are illustrated using energy level diagram as shown in Figure S13.

### Fabrication of flexible transparent displays based on UCNP-incorporated polymer waveguide

**Figure 4:**
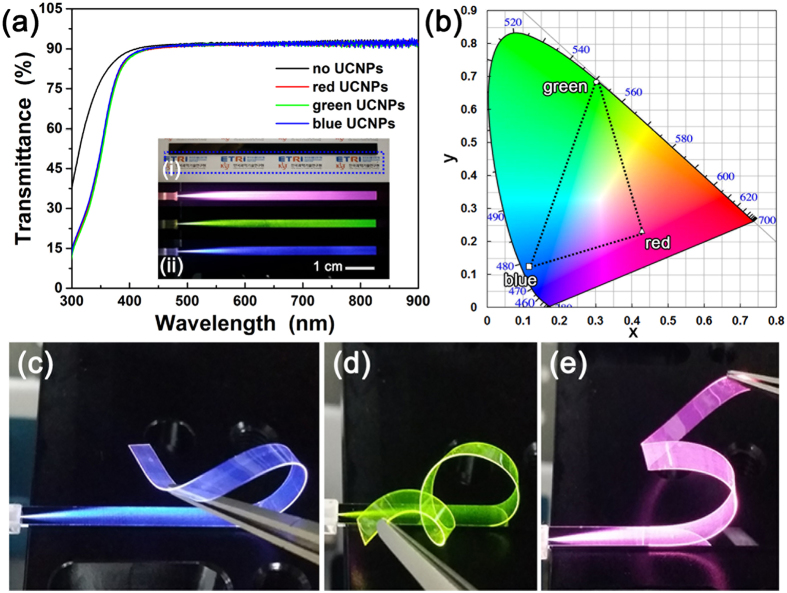
(**a**) Transmittance spectra of stripe-type polymer waveguides (black line: bare polymer waveguide without UCNPs, red line: red-emitting UCNP-incorporated polymer waveguide, green line: green-emitting UCNP-incorporated polymer waveguide, and blue line: blue-emitting UCNP-incorporated polymer waveguide). Inset in (**a**) shows photographs of (i) the fabricated polymer waveguides (bottom: free-standing UCNP-incorporated polymer waveguide detached from the Si substrate, which is enclosed with dotted blue line, and top: as-fabricated UCNP-incorporated polymer waveguide on the Si substrate) and (ii) luminescent polymer waveguides coupled with a 980 nm NIR laser (blue-, green-, and red-emitting C/S UCNP-incorporated polymer waveguides from bottom to top). (**b**) CIE color coordinates of blue-, green-, and red-emitting C/S UCNP solutions (□: blue-emitting C/S UCNPs, ○: green-emitting C/S UCNPs, and Δ: red-emitting C/S UCNPs). Photographs showing the luminescence from severely bended (**c**) blue-, (**d**) green-, and (**e**) red-emitting C/S UCNP-incorporated polymer waveguides, respectively. The logos in (**a**) inset were reprinted with permission from Korea Institute of Science and Technology (KIST) and Electronics and Telecommunications Research Institute (ETRI).

These blue-, green-, and red-emitting C/S UCNPs were incorporated into bisphenol A ethoxylate diacrylate to fabricate polymer waveguide-based flexible transparent display devices. It was reported that bisphenol A ethoxylate diacrylate can be applied to the polymer waveguide core material for transparent thin film displays^23^, and we used bisphenol A ethoxylate diacrylate as a core material constituting polymer waveguides for application to the flexible transparent displays. The C/S UCNP-polymer mixtures also exhibited bright blue, green, and red UCL like the C/S UCNP solutions under the illumination with 980 nm NIR light, as shown in Figure S14. The UC blue-, green-, and red-emitting polymer waveguides were fabricated by spin-coating the blue-, green-, and red-emitting C/S UCNP-incorporated bisphenol A ethoxylate diacrylate core materials on the lower clad material (tetra(ethylene glycol) diacrylate), respectively, followed by formation of tetra(ethylene glycol) diacrylate upper clad. As shown in Fig. 4a, the fabricated C/S UCNP-incorporated polymer waveguides were highly transparent. All the fabricated waveguides (thickness ~38 μm) containing blue-, green-, and red-emitting UCNPs exhibited high transparency with transmittance values of 86.7–93% in visible spectral region (400–800 nm) (Fig. 4a). In particular, the fabricated polymer waveguides exhibited high transmittance over 90% in the spectral range from 443 to 900 nm. Considering the transmittance value (89–93% in visible spectral range) of the bare polymer waveguide without the C/S UCNPs, it should be noted that there was little influence of the incorporation of C/S UCNPs into the polymer waveguides on the reduction of the transparency of the waveguides in visible spectral region. (Also, mechanical property of the polymer substrate was investigated and it was shown in Figure S15.) Due to this high transparency of the UCNP-incorporated polymer waveguides, background letters under the polymer waveguide are clearly seen (Fig. 4a–i). When the fabricated stripe-type polymer waveguides were coupled with a fiber laser emitting 980 nm NIR light, the waveguides exhibited uniform blue, green, and red light, respectively, indicating uniform dispersion of the C/S UCNPs in the bisphenol A ethoxylate diacrylate (Fig. 4a-ii). As shown in Fig. 4b, the blue-, green-, and red-emitting C/S UCNPs exhibited the Commission Internationale de l’Eclairage (CIE) color coordinates of (0.1170, 0.1251), (0.3012, 0.6835), and (0.4290, 0.2329), respectively. Thus, any luminescence color from the waveguide can be generated in the triangle which is formed by connecting the CIE color coordinates of blue-, green-, and red-emitting C/S UCNPs by simply blending three C/S UCNP components. The color space generated by these blue-, green-, and red-emitting C/S UCNPs exhibited relatively narrow color gamut range compared with National Television System Committee (NTSC) color space (~49% NTSC). The narrow color gamut is attributed to the low color purity of the red light from the NaGdF_4_:Yb,Tm/NaGdF_4_:Eu UCNPs and the color gamut can be widened if another efficient red-emitting UCNPs with higher color purity are used. As shown in the scanning electron microscopy (SEM) image of cross-section of the fabricated stripe-type waveguide, the thickness of the fabricated waveguide was observed to be ~38 μm (Figure S16). Because the polymer waveguides are very thin and have flexible nature of the polymer, they are easily bendable and the bended waveguides also exhibited bright and uniform UC blue, green, and red lights like flat waveguides without bending shown in Fig. 4a-ii (see Figure S17). Furthermore, UC blue, green, and red lights are well observed along the severely bended waveguides and there was no discrepancy of UCL between before and after bending (Fig. 4c–e). It is noted that the brightness of the UCL emitted from the fabricated waveguides can be easily enhanced by increasing input laser power (Figure S18).

**Figure 5:**
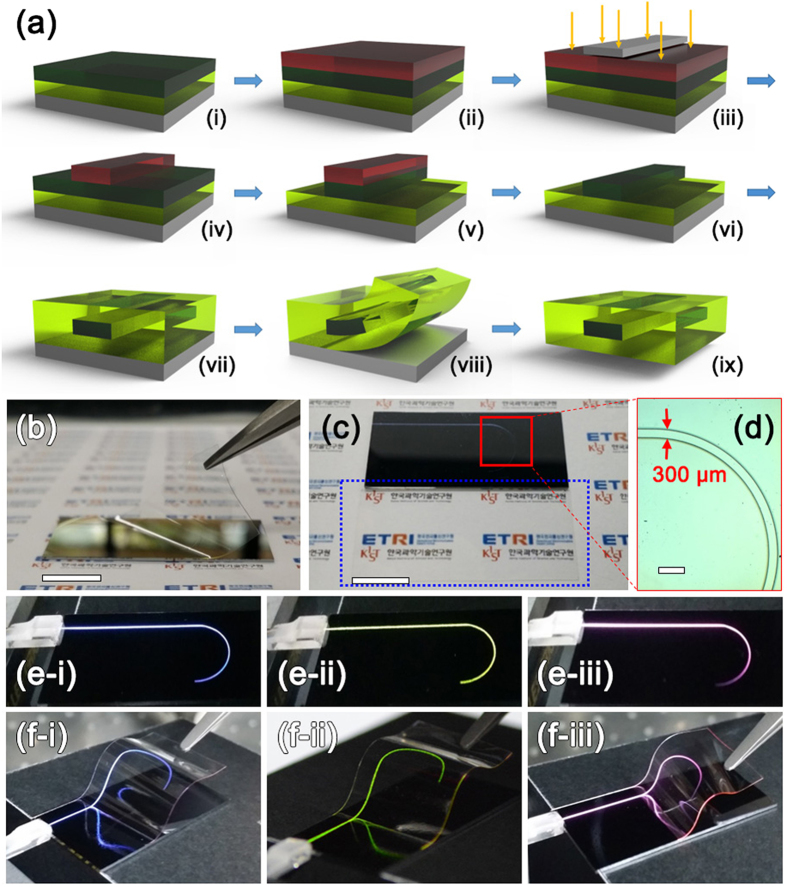
(**a**) Schematic illustration showing the fabrication of patterned C/S UCNP-incorporated polymer waveguide [i: formation of UCNP-incorporated polymer core on the lower clad layer on a Si substrate, ii: formation of photoresist (PR) on the polymer core, iii: UV light exposure using a mask and a UV lamp, iv: patterned PR, v: core patterning through RIE process, vi: removal of PR, vii: formation of upper clad on the patterned core, viii: detachment of a patterned waveguide, and ix: free-standing patterned waveguide]. (**b**) Photograph showing detachment of the fabricated patterned polymer waveguide corresponding to procedure a-viii. (**c**) Photograph of a patterned waveguide on a Si substrate (top) and a free-standing patterned waveguide, which is indicated with dotted blue line (bottom). (**d**) Optical microscope image of selected area (enclosed with red square) of the patterned waveguide. Photographs showing the blue, green, and red UC luminescence from (**e**) the patterned waveguides on Si substrates and (**f**) severely bended patterned waveguides under coupling with an NIR laser. (i: blue-emitting polymer waveguide, ii: green-emitting polymer waveguide, and iii: red-emitting polymer waveguide) Scale bars in (**b**) and (**c**) indicate 10 mm, and scale bar in (**d**) indicates 1  mm. The logos in (**b**) and (**c**) were reprinted with permission from Korea Institute of Science and Technology (KIST) and Electronics and Telecommunications Research Institute (ETRI).

Furthermore, patterned waveguides were constructed through reactive ion etching (RIE) process as depicted in Fig. 5a (also see Figure S19). First, the lower clad layer (tetra(ethylene glycol) diacrylate) was spin-coated on a Si wafer and UV-cured (2 kW, 5 min), and the core layer (C/S UCNP-incorporated bisphenol A ethoxylate diacrylate) was spin-coated on the lower clad layer and UV-cured (2 kW, 5 min) (Fig. 5a–i). Second, optical waveguide was patterned by photolithography and RIE process (Fig. 5a-ii–5a-vi). Third, the upper clad layer was spin-coated on the patterned optical waveguide and UV-cured at 2 kW for 5 min (Fig. 5a-vii). Finally, the patterned optical waveguide was detached from the Si wafer (Fig. 5a-viii and 5a-ix). The patterned waveguide-based display devices were also highly transparent as shown in Fig. 5b and c. Patterned letter “J” of the free-standing waveguide is not distinguishable to the naked eyes although the pattern is evidently seen in the optical microscope image shown in Fig. 5d. However, blue, green, and red capital “J”s were distinctly observed owing to UCL along the patterned optical waveguides when the waveguides were coupled with 980 nm NIR light (Fig. 5e). The color of the patterned letter was easily tailored by adjusting the kinds of the incorporated UCNPs into the core polymer constituting the waveguides. Since these patterned waveguide-based display devices were also flexible, UCL images can be displayed from the largely curved waveguides. While the waveguides were severely bended, blue, green, and red UCL images (i.e., blue, green, and red capital “J”s) were clearly seen without any difference between UCL uniformities before and after bending (Fig. 5f). It is worthy to note that all these UCL images from the patterned waveguides are clearly seen under ambient indoor light condition.

## Conclusion

In summary, highly transparent and flexible display devices based on C/S UCNP-incorporated polymer waveguide have been demonstrated. The C/S-structured Li(Gd,Y)F_4_:Yb,Er/LiYF_4_ and Li(Gd,Y)F_4_:Yb,Tm/LiYF_4_ UCNPs were successfully synthesized and they showed strong UCL intensities enhanced by the factors of 3.3 and 2.0 compared with Li(Gd,Y)F_4_:Yb,Er, and Li(Gd,Y)F_4_:Yb,Tm cores, respectively. As a result, they exhibited bright green and blue emission under illumination with 980 nm NIR light, respectively. The synthesized NaGdF_4_:Yb,Tm/NaGdF_4_:Eu C/S UCNPs showed red emission via ^5^D_0_ → ^7^F_2_ transition in Eu^3+^ in the shell through energy migration UC process under excitation with 980 nm NIR light. These blue-, green-, and red-emitting C/S UCNPs were incorporated into the bisphenol A ethoxylate diacrylate to fabricate flexible transparent waveguides. The polymer waveguide-based devices were highly transparent (transmittance > 90% in the spectral range of 443–900 nm). The flexible transparent monochromatic display devices were fabricated by patterning polymer waveguide through RIE process. The patterned polymer waveguide-based devices exhibited bright blue-, green-, and red-colored letters under coupling with a 980 nm NIR laser, irrespective of severe bending. These results can be a cornerstone to realize multicolor-emitting flexible transparent display devices utilizing NIR light.

## Methods

### Materials

To synthesize blue-, green-, and red-emitting UCNPs, LiOH∙H_2_O (99.995%), NaOH (99.99%), GdCl_3_∙6H_2_O (99%), YCl_3_∙6H_2_O (99.99%), YbCl_3_∙6H_2_O (99.9%), ErCl_3_∙6H_2_O (99.9%), TmCl_3_∙6H_2_O (99.99%), EuCl_3_∙6H_2_O (99.99%), NH_4_F (≥99.99%), oleic acid (OA, technical grade 90%), and 1-octadecene (ODE, technical grade 90%) were purchased from Aldrich and they were used without further purification. Sodium oleate (>97%) was obtained from TCI. To fabricate polymer waveguide-based flexible transparent displays, bisphenol A ethoxylate diacrylate (average molecular weight ~468) and tetra(ethylene glycol) diacrylate (molecular weight 302.32) were obtained from Aldrich.

### Syntheses of the Li(Gd,Y)F_4_:Yb,Er and Li(Gd,Y)F_4_:Yb,Tm UCNPs

For the synthesis of Li(Gd,Y)F_4_:Yb,Er UCNPs, rare earth oleate (RE(oleate)_3_, RE = Gd, Y, Yb, and Er) complexes were firstly prepared by adopting previously reported method by Hyeon’s group^44^. Then 1 mmol of RE(oleate)_3_ complexes (RE = Gd (35%), Y (45%), Yb (18%), and Er (2%)) were loaded into three-neck flask containing mixed solvents of OA (10.5 mL) and ODE (10.5 mL). The mixture was heated to 150 °C and maintained for 40 min to yield a transparent solution. After the reaction solution was cooled to 50 °C, a methanol (MeOH) solution (10 mL) containing LiOH·H_2_O (2.5 mmol) and NH_4_F (4 mmol) was injected into the reaction flask and then the reaction mixture was stirred for 40 min. After the MeOH was removed, the solution was heated to 320 °C and maintained for 90 min under Ar atmosphere. The as-synthesized Li(Gd,Y)F_4_:Yb,Er UCNPs were washed several times with ethanol (EtOH) and dispersed into chloroform. In the case of the synthesis of Li(Gd,Y)F_4_:Yb,Tm UCNPs, 1 mmol of RE(oleate)_3_ complexes (RE = Gd (34.5%), Y (40%), Yb (25%), and Tm (0.5%)) were used as precursors and other synthetic procedures were the same as those for Li(Gd,Y)F_4_:Yb,Er UCNPs.

### Synthesis of the Li(Gd,Y)F_4_:Yb,Er/LiYF_4_ and Li(Gd,Y)F_4_:Yb,Tm/LiYF_4_ C/S UCNPs

To synthesize green-emitting C/S UCNPs, YCl_3_·6H_2_O (1 mmol) were dissolved in mixed solvents of OA (10.5 mL) and ODE (10.5 mL) by heat-treatment at 150 °C for 40 min. After the reaction mixture was cooled to 80 °C, 10 mL of Li(Gd,Y)F_4_:Yb,Er UC core chloroform solution was injected into the reaction flask, and then 10 mL of MeOH solution containing LiOH·H_2_O (2.5 mmol) and NH_4_F (4 mmol) was added to the reaction solution. After stirring at 50 °C for 40 min, MeOH was removed and the reaction solution was heated to 300 °C and maintained for 110 min under Ar atmosphere. The as-synthesized C/S UCNPs were washed with EtOH several times and dispersed in 10 mL of chloroform. For the synthesis of Li(Gd,Y)F_4_:Yb,Tm/LiYF_4_ C/S UCNPs, 10 mL of Li(Gd,Y)F_4_:Yb,Tm UC core chloroform solution was used and other procedures were the same as those for the synthesis of Li(Gd,Y)F_4_:Yb,Er/LiYF_4_ C/S UCNPs.

### Synthesis of the NaGdF_4_:Yb,Tm UCNPs

The NaGdF_4_:Yb,Tm UCNPs were synthesized by slightly modifying the method reported by Liu’s group^33^. GdCl_3_·6H_2_O (0.5 mmol), YbCl_3_·6H_2_O (0.49 mmol), and TmCl_3_·6H_2_O (0.01 mmol) were loaded into the three-neck flask containing the mixed solvents of OA (6 mL) and ODE (15 mL). The mixed solution was heated to 150 °C and maintained for 40 min. After the reaction mixture was cooled to 50 °C, 10 mL of MeOH solution containing NaOH (2.5 mmol) and NH_4_F (4 mmol) was added to the reaction solution. After stirring at 50 °C for 40 min, MeOH was removed and the reaction solution was heated to 310 °C and maintained for 90 min under Ar atmosphere. The as-synthesized UCNPs were washed with EtOH several times and dispersed in 10 mL of chloroform.

### Synthesis of the NaGdF_4_:Yb,Tm/NaGdF_4_:Eu C/S UCNPs

To synthesize red-emitting C/S UCNPs, GdCl_3_·6H_2_O (0.85 mmol) and EuCl_3_·6H_2_O (0.15 mmol) were dissolved in mixed solvents of OA (6 mL) and ODE (15 mL) by heat-treatment at 150 °C for 40 min. After the reaction mixture was cooled to 80 °C, 10 mL of NaGdF_4_:Yb,Tm UC core chloroform solution was injected into the reaction flask, and then 10 mL of MeOH solution containing NaOH (2.5 mmol) and NH_4_F (4 mmol) was added to the reaction solution. After stirring at 50 °C for 40 min, MeOH was removed and the reaction solution was heated to 310 °C and maintained for 110 min under Ar atmosphere. The as-synthesized C/S UCNPs were washed with EtOH several times and dispersed in 10 mL of chloroform.

### Fabrication of the flexible transparent display devices based on C/S UCNP-incorporated polymer waveguides

To fabricate polymer waveguide-based flexible transparent display devices, the synthesized C/S UCNPs were first incorporated into bisphenol A ethoxylate diacrylate (refractive index, n_a_ = 1.5647 at 632 nm) polymer core (0.12 wt%). The C/S UCNP-incorporated polymer was spin-coated on the lower clad (tetra(ethylene glycol) diacrylate, n_a_ = 1.5031 at 632 nm), which was spin-coated on a Si wafer at 500 rpm for 20 s and UV-cured at 2 kW for 5 min, at 3700 rpm for 30 s and UV-cured at 2 kW for 5 min. Then, patterned optical waveguide was formed by photolithography and RIE process. Next, the upper clad was formed by spin-coating tetra(ethylene glycol) diacrylate on the patterned optical waveguide and UV-cured under the same condition as that for the formation of the lower clad layer. Finally, the blue-, green-, and red-emitting patterned waveguides were detached from the Si wafers.

### Characterization

All PL spectra of the blue-, green-, and red-emitting UCNPs were collected with a Hitachi F-7000 spectrophotometer. The TEM images of the UCNPs were obtained by using an FEI Tecnai F20 G^2^ transmission electron microscope operating at 200 kV and EDS maps on the C/S UCNPs were obtained on an FEI Talos F200X scanning transmission electron microscope (S/TEM) operating at 200 kV. The crystal structures of the synthesized UCNPs were investigated by XRD using a Bruker D8 ADVANCE diffractometer with Cu K_α_ radiation under the condition of 40 kV and 40 mA. The HR-STEM images were obtained with an aberration-corrected FEI Titan 80–300 S/TEM operating at 300 kV. Transmittance of the fabricated devices were measured by using a Shimadzu UV-2600 spectrophotometer.

## Additional Information

**How to cite this article:** Park, B.J. *et al*. Flexible transparent displays based on core/shell upconversion nanophosphor-incorporated polymer waveguides. *Sci. Rep.* **7**, 45659; doi: 10.1038/srep45659 (2017).

**Publisher's note:** Springer Nature remains neutral with regard to jurisdictional claims in published maps and institutional affiliations.

## Supplementary Material

Supplementary Information

## References

[b1] Auzel F. Upconversion and Anti-Stokes Processes with f and d Ions in Solids. Chem. Rev. 104, 139–174 (2004). 14719973 10.1021/cr020357g

[b2] Wang F. & Liu X. Recent advances in the chemistry of lanthanide-doped upconversion nanocrystals. Chem. Soc. Rev. 38, 976–989 (2009). 19421576 10.1039/b809132n

[b3] Chen G. . Light upconverting core-shell nanostructures: nanophotonic control for emerging applications. Chem. Soc. Rev. 44, 1680–1713 (2015). 25335878 10.1039/c4cs00170b

[b4] Boyer J.-C. . Synthesis of Colloidal Upconverting NaYF_4_ Nanocrystals Doped with Er^3+^, Yb^3+^ and Tm^3+^, Yb^3+^ via Thermal Decomposition of Lanthanide Trifluoroacetate Precursors. J. Am. Chem. Soc. 128, 7444–7445 (2006). 16756290 10.1021/ja061848b

[b5] Gai S. . Recent Progress in Rare Earth Micro/Nanocrystals: Soft Chemical Synthesis, Luminescent Properties, and Biomedical Applications. Chem. Rev. 114, 2343–2389 (2014). 24344724 10.1021/cr4001594

[b6] Li Z. . Multicolor Core/Shell-Structured Upconversion Fluorescent Nanoparticles. Adv. Mater. 20, 4765–4769 (2008).

[b7] Mai H.-X. . High-Quality Sodium Rare-Earth Fluoride Nanocrystals: Controlled Synthesis and Optical Properties. J. Am. Chem. Soc. 128, 6426–6436 (2006). 16683808 10.1021/ja060212h

[b8] Wang F. . Simultaneous phase and size control of upconversion nanocrystals through lanthanide doping. Nature 463, 1061–1065 (2010). 20182508 10.1038/nature08777

[b9] Zhou J. . Upconversion Luminescent Materials: Advances and Applications. Chem. Rev. 115, 395–465 (2015). 25492128 10.1021/cr400478f

[b10] Weissleder R. A clearer vision for *in vivo* imaging. Nat. Biotechnol. 19, 316–317 (2001). 11283581 10.1038/86684

[b11] Xiong L.-Q. . Synthesis, characterization, and *in vivo* targeted imaging of amine-functionalized rare-earth up-converting nanophosphors. Biomaterials 30, 5592–5600 (2009). 19564039 10.1016/j.biomaterials.2009.06.015

[b12] Chatterjee D. K. . Upconversion fluorescence imaging of cells and small animals using lanthanide doped nanocrystals. Biomaterials 29, 937–943 (2008). 18061257 10.1016/j.biomaterials.2007.10.051

[b13] Wu S. . Non-blinking and photostable upconverted luminescence from single lanthanide-doped nanocrystals. Proc. Natl. Acad. Sci. USA 106, 10917–10921 (2009). 19541601 10.1073/pnas.0904792106 PMC2698891

[b14] Zeng S. . Simultaneous Realization of Phase/Size Manipulation, Upconversion Luminescence Enhancement, and Blood Vessel Imaging in Multifunctional Nanoprobes Through Transition Metal Mn^2+^ Doping. Adv. Funct. Mater. 24, 4051–4059 (2014).

[b15] Park Y. I. . Nonblinking and Nonbleaching Upconverting Nanoparticles as an Optical Imaging Nanoprobe and T1 Magnetic Resonance Imaging Contrast Agent. Adv. Mater. 21, 4467–4471 (2009).

[b16] Zhao L. . Stem Cell Labeling using Polyethylenimine Conjugated (α-NaYbF_4_:Tm^3+^)/CaF_2_ Upconversion Nanoparticles. Theranostics 3, 249–257 (2013). 23606911 10.7150/thno.5432 PMC3630525

[b17] Sun Y. . Upconversion Nanophosphors NaLuF_4_:Yb,Tm for Lymphatic Imaging *In Vivo* by Real-Time Upconversion Luminescence Imaging under Ambient Light and High-Resolution X-ray CT. Theranostics 3, 346–353 (2013). 23650481 10.7150/thno.5137 PMC3645060

[b18] Downing E. . A Three-Color, Solid-State, Three-Dimensional Display. Science 273, 1185–1189 (1996).

[b19] Kador L. A Three-Color, Three-Dimensional, Solid-State Display. Adv. Mater. 9, 83–85 (1997).

[b20] Deng R. . Temporal full-colour tuning through non-steady-state upconversion. Nat. Nanotechnol. 10, 237–242 (2015). 25599189 10.1038/nnano.2014.317

[b21] Rapaport A. . H. Review of the Properties of Up-Conversion Phosphors for New Emissive Displays. J. Display Technol. 2, 68–78 (2006).

[b22] Okuda Y. & Fujieda I. Polymer waveguide technology for flexible display applications. Proc. SPIE 8280, Advances in Display Technologies II, 82800W (2012).

[b23] Park S. . Thin film display based on polymer waveguides. Opt. Express 22, 23433–23438 (2014). 25321812 10.1364/OE.22.023433

[b24] Na H. . Facile synthesis of intense green light emitting LiGdF_4_:Yb,Er-based upconversion bipyramidal nanocrystals and their polymer composites. Nanoscale 6, 7461–7468 (2014). 24882742 10.1039/c4nr00857j

[b25] Park H. K. . Toward scatter-free phosphors in white phosphor-converted light-emitting diodes. Opt. Express 20, 10218–10228 (2012). 22535113 10.1364/OE.20.010218 PMC3482912

[b26] Wang F. . Direct Evidence of a Surface Quenching Effect on Size-Dependent Luminescence of Upconversion Nanoparticles. Angew. Chem. Int. Ed. 49, 7456–7460 (2010). 10.1002/anie.201003959 20809558

[b27] Jang H. S. . Bright dual-mode green emission from selective set of dopant ions in β-Na(Y,Gd)F_4_:Yb,Er/β-NaGdF_4_:Ce,Tb core/shell nanocrystals. Opt. Express 20, 17107–17118 (2012).

[b28] Su Q. . The Effect of Surface Coating on Energy Migration-Mediated Upconversion. J. Am. Chem. Soc. 134, 20849–20857 (2012). 23210614 10.1021/ja3111048

[b29] Chen X. . Photon upconversion in core-shell nanoparticles. Chem. Soc. Rev. 44, 1318–1330 (2015). 25058157 10.1039/c4cs00151f

[b30] Watanabe S. . 3D Micromolding of Arrayed Waveguide Gratings on Upconversion Luminescent Layers for Flexible Transparent Displays without Mirrors, Electrodes, and Electric Circuits. Adv. Funct. Mater. 25, 4390–4396 (2015).

[b31] Mahalingam V. . A. Colloidal Tm^3+^/Yb^3+^-Doped LiYF_4_ Nanocrystals: Multiple Luminescence Spanning the UV to NIR Regions via Low-Energy Excitation. Adv. Mater. 21, 4025–4028 (2009).

[b32] Wang J. . Lanthanide-doped LiYF_4_ nanoparticles: Synthesis and multicolor upconversion tuning. C. R. Chim. 13, 731–736 (2010).

[b33] Wang F. . Tuning upconversion through energy migration in core-shell nanoparticles. Nat. Mater. 10, 968–973 (2011). 22019945 10.1038/nmat3149

[b34] Boyer J.-C. & van Veggel F. C. J. M. Absolute quantum yield measurements of colloidal NaYF_4_:Er^3+^,Yb^3+^ upconverting nanoparticles. Nanoscale 2, 1417–1419 (2010). 20820726 10.1039/c0nr00253d

[b35] Schäfer H. . Synthesis and Optical Properties of KYF_4_/Yb, Er Nanocrystals, and their Surface Modification with Undoped KYF_4_. Adv. Funct. Mater. 18, 2913–2918 (2008).

[b36] Huang P. . Lanthanide-Doped LiLuF_4_ Upconversion Nanoprobes for the Detection of Disease Biomarkers. Angew. Chem. Int. Ed. 53, 1252–1257 (2014). 10.1002/anie.201309503 24436151

[b37] Du Y.-P. . Optically active uniform potassium and lithium rare earth fluoride nanocrystals derived from metal trifluroacetate precursors. Dalton Trans., 8574–8581 (2009). 19809734 10.1039/b909145a

[b38] Kim S. Y. . Direct observation of core/double-shell architecture of intense dual-mode luminescent tetragonal bipyramidal nanophosphors. Nanoscale 8, 10049–10058 (2016). 26729043 10.1039/c5nr05722a

[b39] Guo H. . Seed-mediated synthesis of NaYF_4_:Yb,Er/NaGdF_4_ nanocrystals with improved upconversion fluorescence and MR relaxivity. Nanotechnology 21, 125602 (2010). 20182011 10.1088/0957-4484/21/12/125602

[b40] He M. . Dual Phase-Controlled Synthesis of Uniform Lanthanide-Doped NaGdF_4_ Upconversion Nanocrystals Via an OA/Ionic Liquid Two-Phase System for *In Vivo* Dual-Modality Imaging. Adv. Funct. Mater. 21, 4470–4477 (2011).

[b41] Wen H. . Upconverting Near-Infrared Light through Energy Management in Core–Shell–Shell Nanoparticles. Angew. Chem. Int. Ed. 52, 13419–13423 (2013). 10.1002/anie.201306811 24132883

[b42] Kim S. Y. . A Strategy to enhance Eu^3+^ emission from LiYF_4_:Eu nanophosphors and green-to-orange multicolor tunable, transparent nanophosphor-polymer composites. Sci. Rep. 5, 7866 (2015). 25597900 10.1038/srep07866 PMC4297990

[b43] Liu Y. . A Strategy to Achieve Efficient Dual-Mode Luminescence of Eu^3+^ in Lanthanides Doped Multifunctional NaGdF_4_ Nanocrystals. Adv. Mater. 22, 3266–3271 (2010). 20533416 10.1002/adma.201000128

[b44] Park J. . Ultra-large-scale syntheses of monodisperse nanocrystals. Nat. Mater. 3, 891–895 (2004). 15568032 10.1038/nmat1251

