# Supplementary material for: Flexible transparent displays based on core/shell upconversion nanophosphor-incorporated polymer waveguides
Source: Sci Rep. 2017 Apr 3;7:45659. doi: 10.1038/srep45659 (PMC5377360; doi:10.1038/srep45659)
Supplement: Supplementary Information [file srep45659-s1.pdf]

Supplementary Information

Flexible transparent displays based on core/shell upconversion nanophosphor-incorporated polymer waveguides

Bong Je Park,[1,†] A-Ra Hong,[2,3,†] Suntak Park,[1] Ki-Uk Kyung,[1] Kwangyeol Lee[3] & Ho Seong Jang[2,4,*]

[1]Electronics and Telecommunications Research Institute (ETRI), 218 Gajeong-ro, Yuseong-gu, Daejeon 34129, Republic of Korea

[2]Materials Architecturing Research Center, Korea Institute of Science and Technology, 5, Hwarang-ro 14-gil, Seongbuk-gu, Seoul 02792, Republic of Korea

[3]Department of Chemistry, Korea University, 145 Anam-ro, Seongbuk-gu, Seoul 136-701, Republic of Korea

[4]Department of Nanomaterials Science and Engineering, Korea University of Science and Technology, 218 Gajeong-ro, Yuseong-gu, Daejeon 34113, Republic of Korea

To whom correspondence should be addressed.

Postal Address: Dr. H. S. Jang

Materials Architecturing Research Center

KIST (Korea Institute of Science and Technology)

5, Hwarang-ro 14-gil, Seongbuk-gu, Seoul, 02792 (Republic of Korea)

Tel: +82-2-958-5263, Fax: +82-2-958-5599

e-mail: msekorea@kist.ac.kr

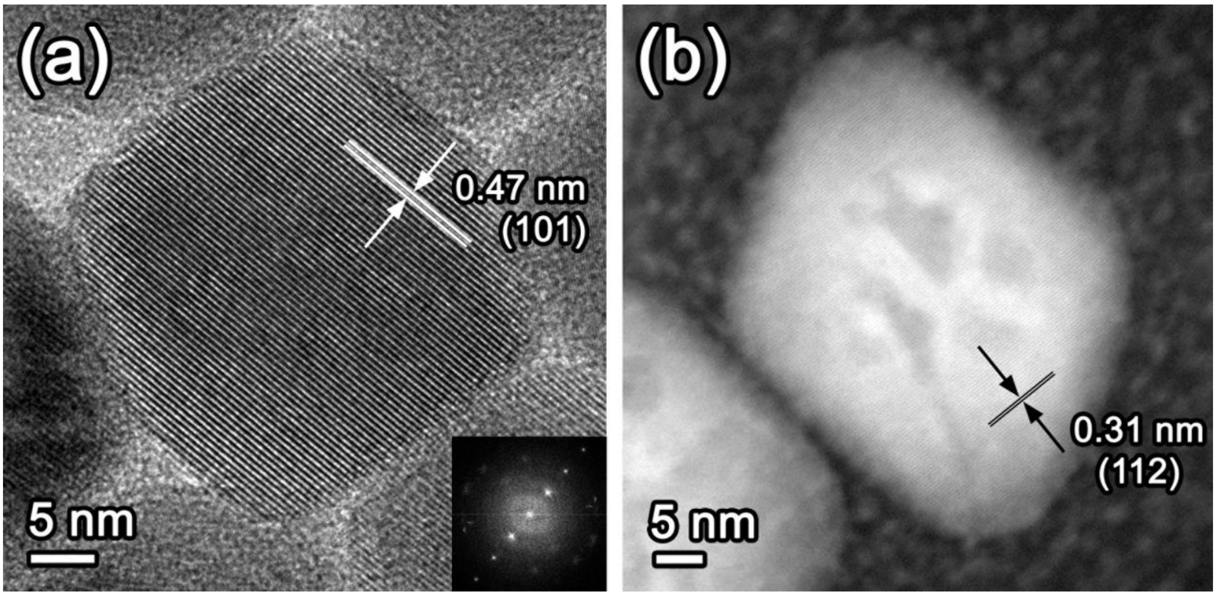

Figure S1. (a) HR-TEM image and (b) HR-STEM image of Li(Gd,Y)F_4:Yb,Er UCNPs. Inset shows fast Fourier transform (FFT) pattern for the HR-TEM image.

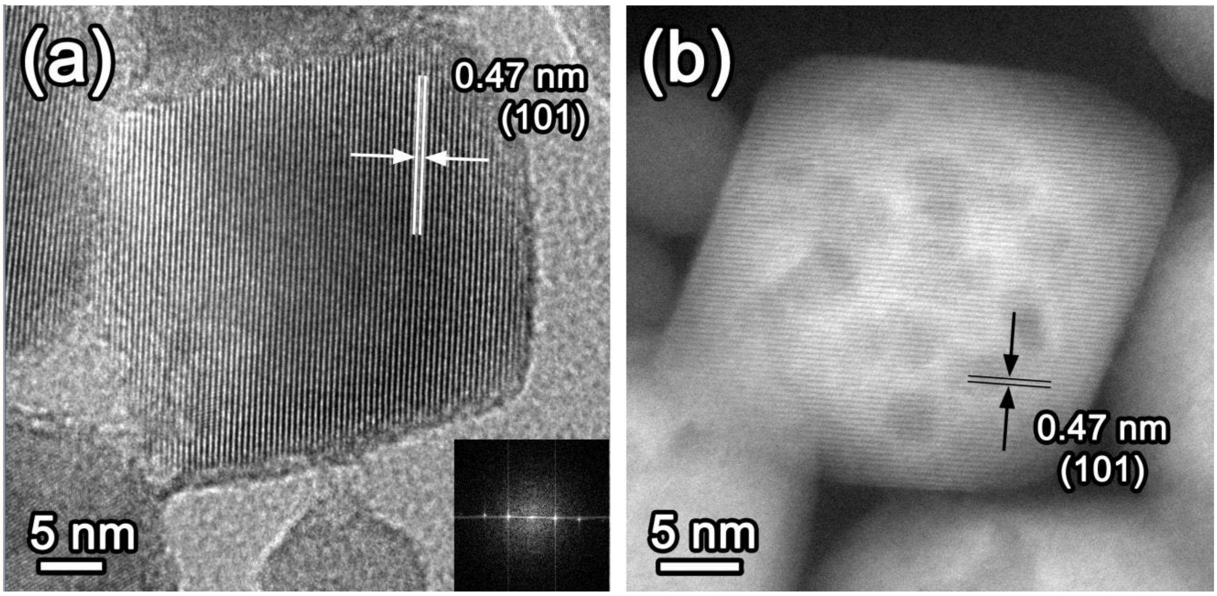

Figure S2. (a) HR-TEM image and (b) HR-STEM image of Li(Gd,Y)F_4:Yb,Er/LiYF_4 C/S UCNPs. Inset shows FFT pattern for the HR-TEM image.

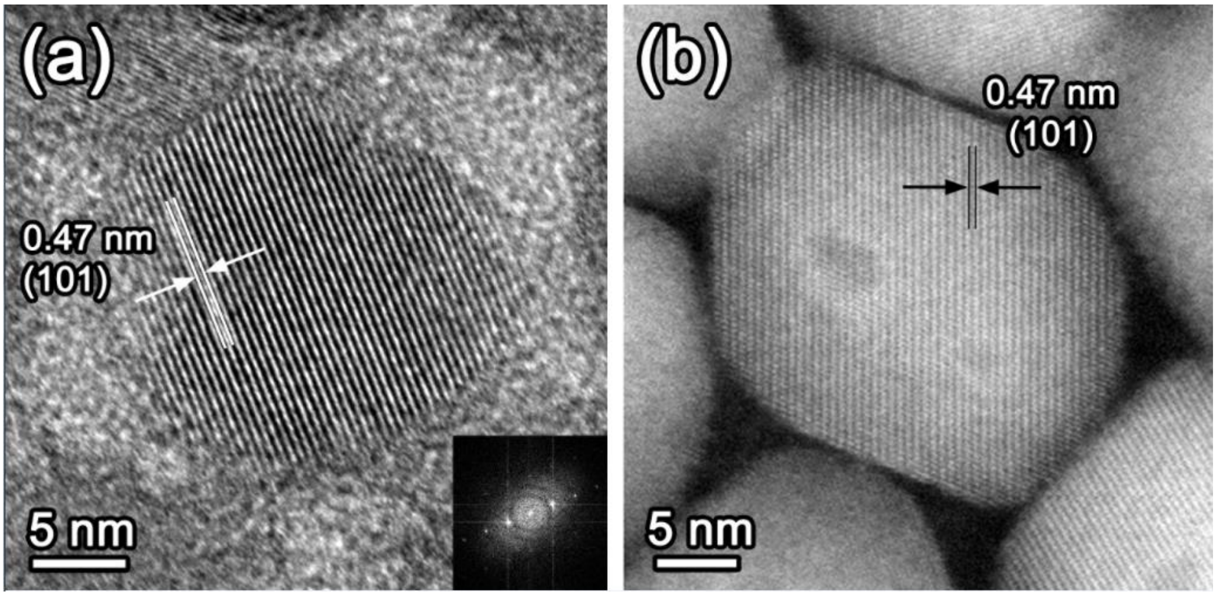

Figure S3. (a) HR-TEM image and (b) HR-STEM image of Li(Gd,Y)F_4:Yb,Tm UCNPs. Inset shows FFT pattern for the HR-TEM image.

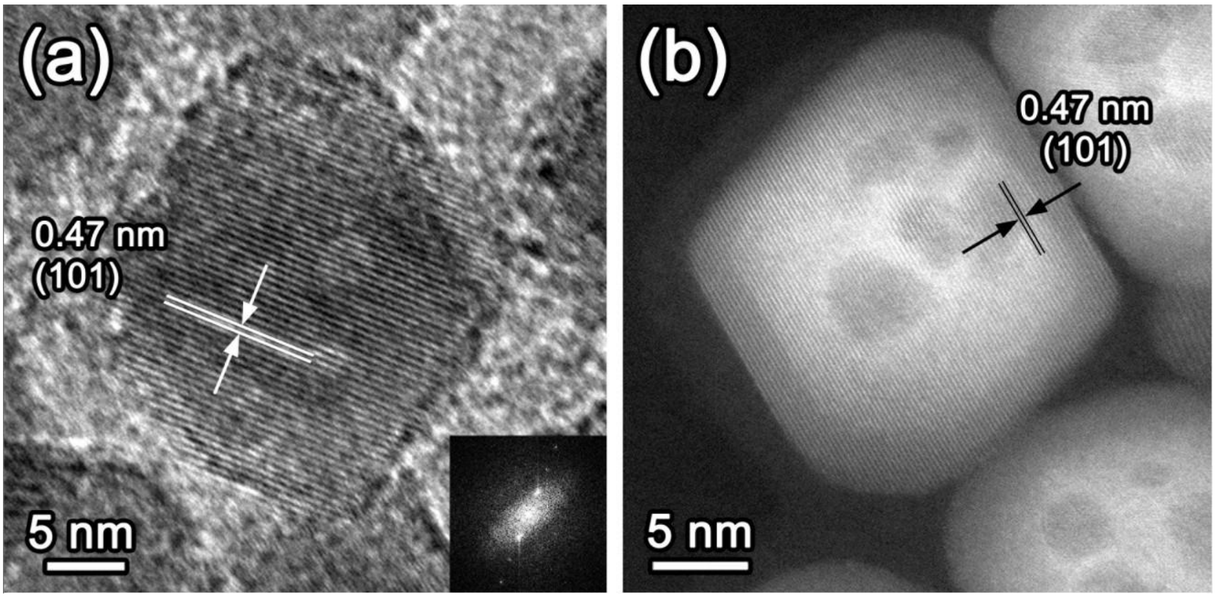

Figure S4. (a) HR-TEM image and (b) HR-STEM image of Li(Gd,Y)F_4:Yb,Tm/LiYF_4 C/S UCNPs. Inset shows FFT pattern for the HR-TEM image.

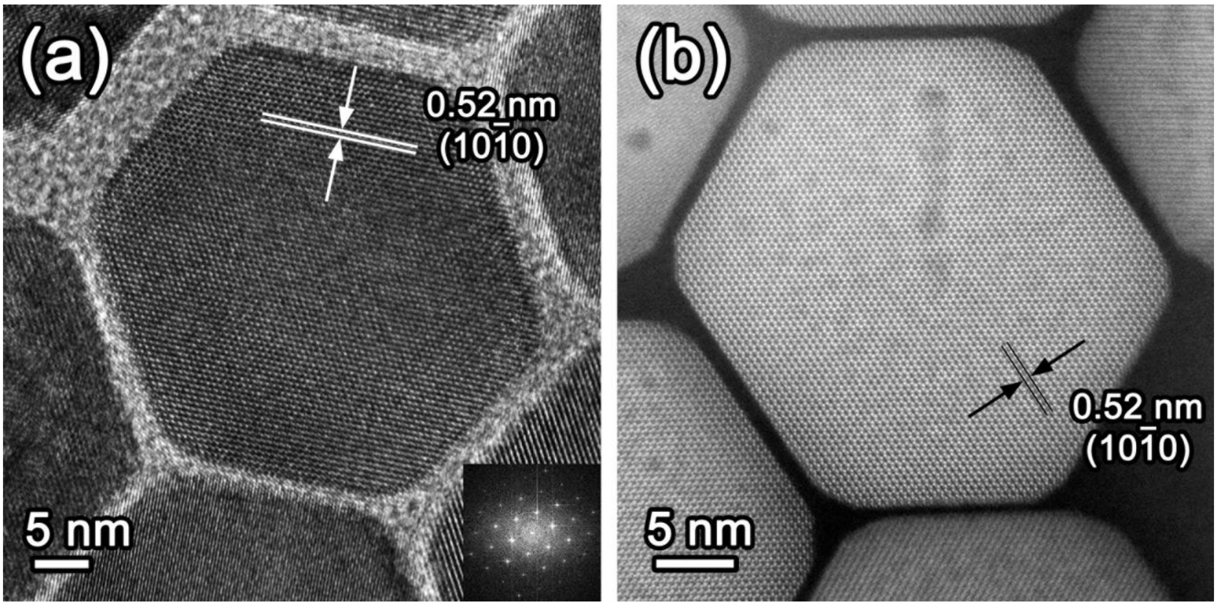

Figure S5. (a) HR-TEM image and (b) HR-STEM image of $NaGdF_4$:Yb,Tm UCNPs. Inset shows FFT pattern for the HR-TEM image.

.

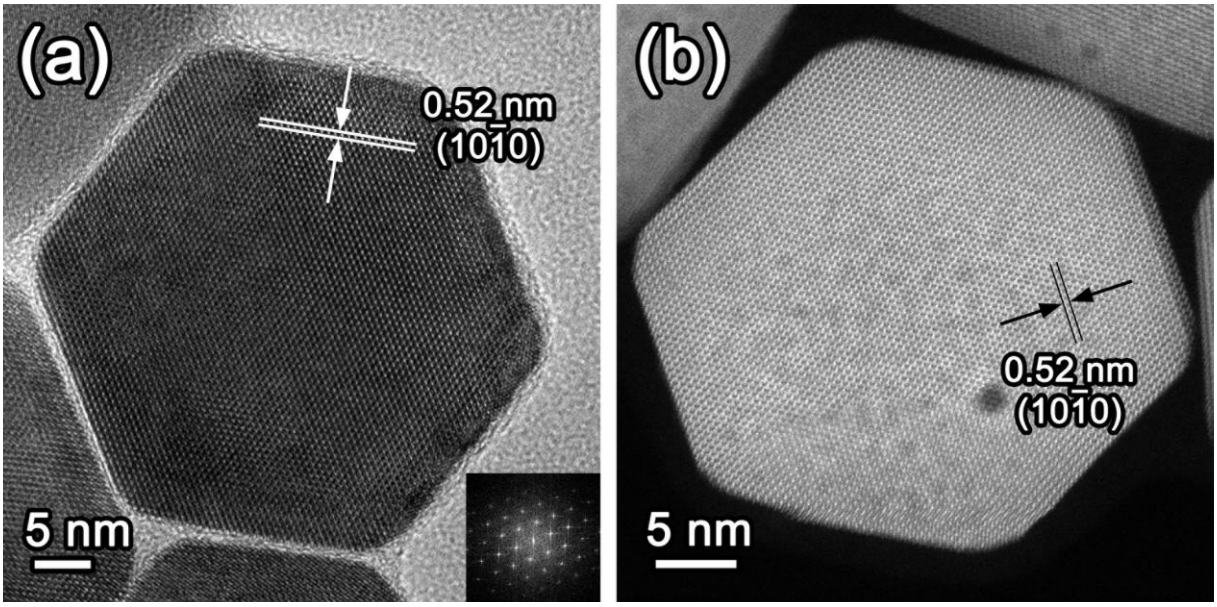

Figure S6. (a) HR-TEM image and (b) HR-STEM image of $NaGdF_4$:Yb,Tm/$NaGdF_4$:Eu C/S UCNPs. Inset shows FFT pattern for the HR-TEM image.

.

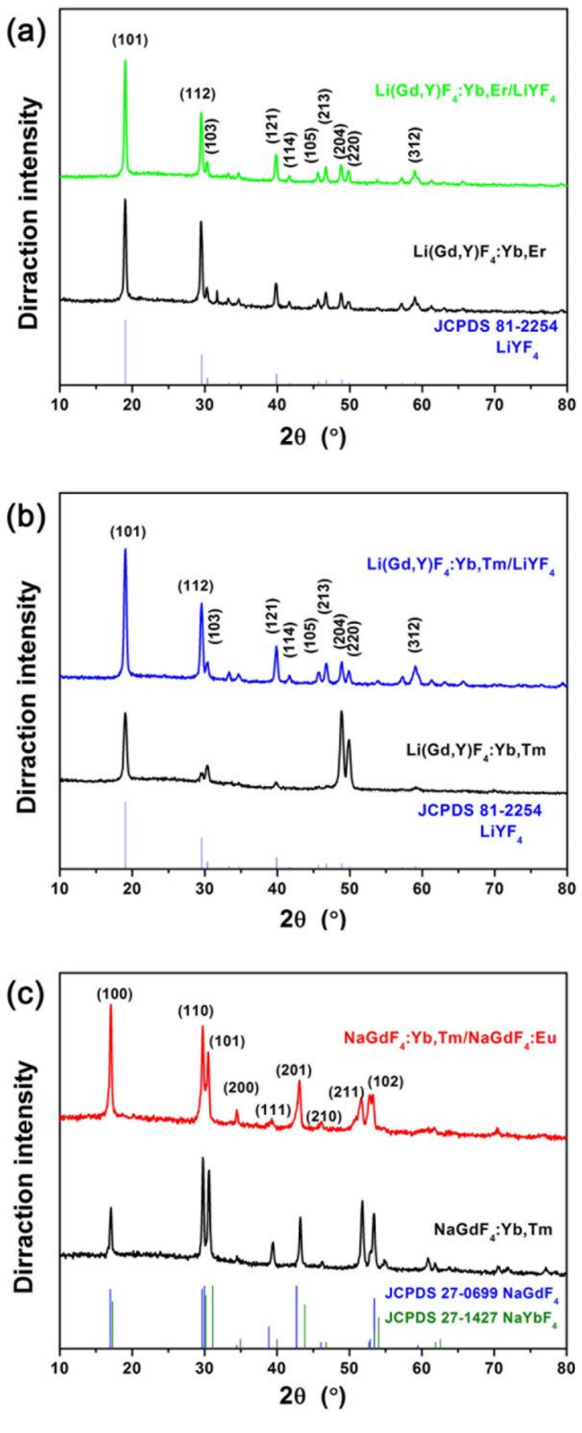

Figure S7. XRD patterns of (a) Li(Gd,Y)F_4:Yb,Er and Li(Gd,Y)F_4:Yb,Er/LiYF_4, (b) Li(Gd,Y)F_4:Yb,Tm and Li(Gd,Y)F_4:Yb,Tm/LiYF_4, and (c) NaGdF_4:Yb,Tm and NaGdF_4:Yb,Tm/NaGdF_4:Eu UCNPs.

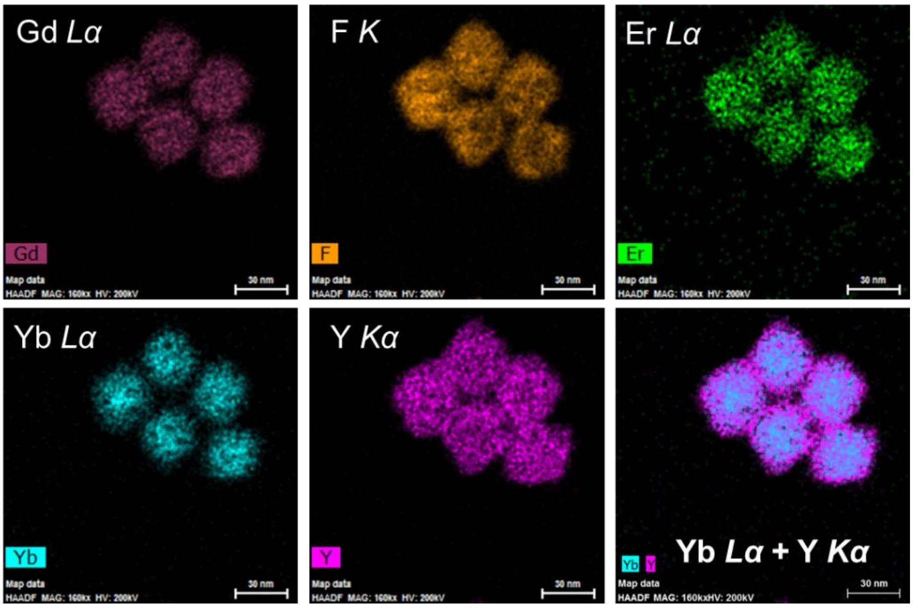

Figure S8. EDS maps of Gd *Lα*, F *K*, Er *Lα*, Yb *Lα*, and Y *Kα* from Li(Gd,Y)F_4:Yb,Er/LiYF_4 C/S UCNPs. The composite EDS map of the C/S UCNPs was produced by superposing Yb *Lα* (cyan) and Y *Kα* (magenta) maps.

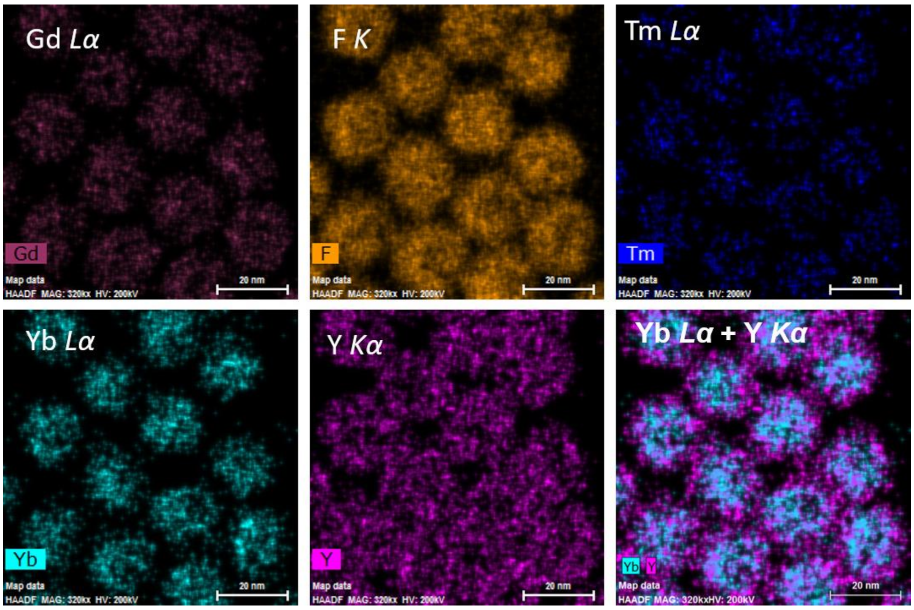

Figure S9. EDS maps of Gd *Lα*, F *K*, Tm *Lα*, Yb *Lα*, and Y *Kα* from Li(Gd,Y)F_4:Yb,Tm/LiYF_4 C/S UCNPs. The composite EDS map of the C/S UCNPs was produced by superposing Yb *Lα* (cyan) and Y *Kα* (magenta) maps.

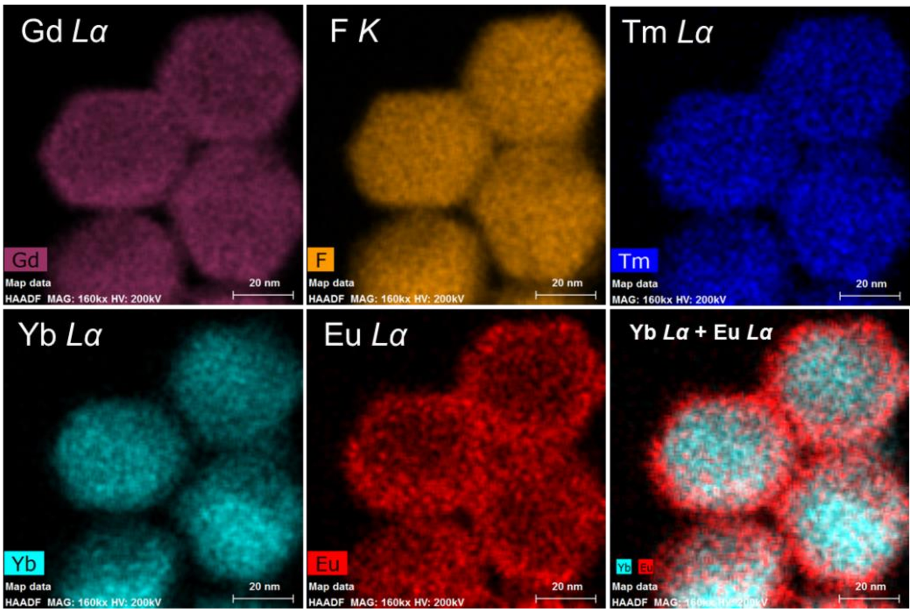

Figure S10. EDS maps of Gd *Lα*, F *K*, Tm *Lα*, Yb *Lα*, and Eu *Lα* from $NaGdF_4$:Yb,Tm/$NaGdF_4$:Eu C/S UCNPs. The composite EDS map of the C/S UCNPs was produced by superposing Yb *Lα* (cyan) and Eu *Lα* (red) maps.

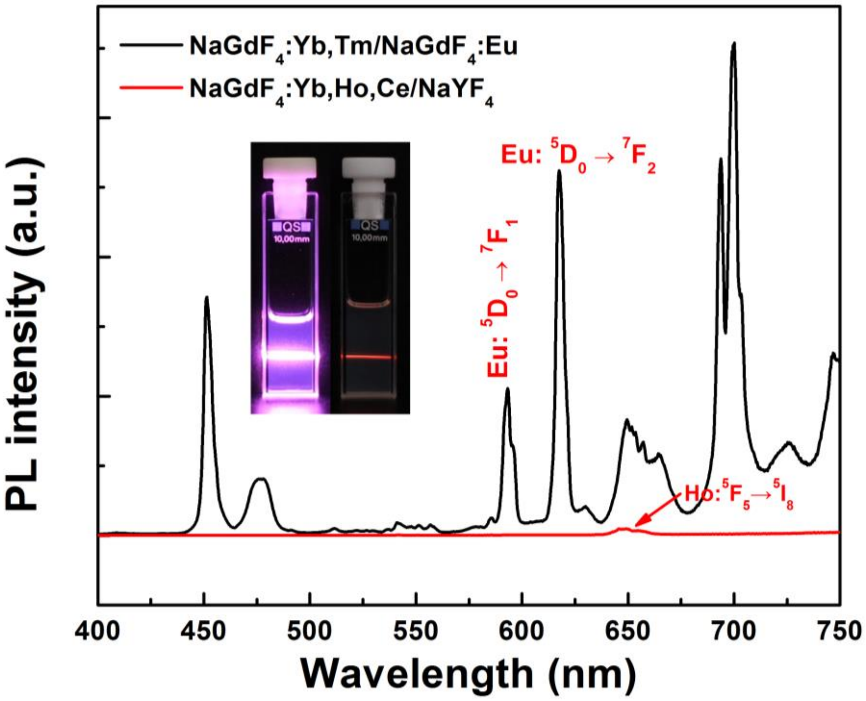

Figure S11. PL spectra of $NaGdF_4$:Yb,Tm/$NaGdF_4$:Eu (black line) and $NaGdF_4$:Yb,Ho,Ce/$NaYF_4$ (red line) UCNPs under excitation with 980 nm NIR light. Inset shows the photograph showing the luminescence from the $NaGdF_4$:Yb,Tm/$NaGdF_4$:Eu (left) and $NaGdF_4$:Yb,Ho,Ce/$NaYF_4$ (right) UCNP solutions under the same excitation condition with a 980 nm NIR laser.

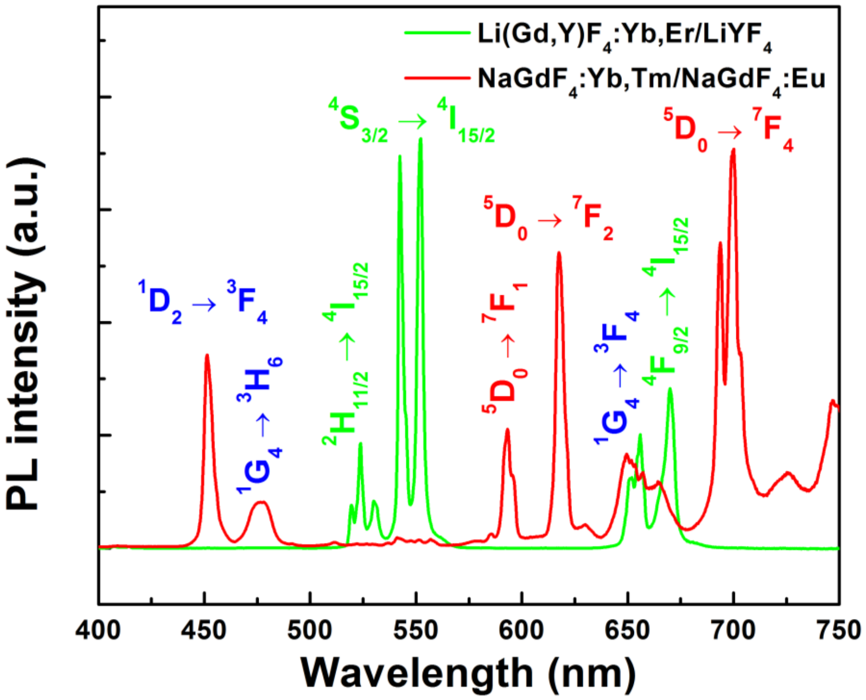

Figure S12. PL spectra of Li(Gd,Y)F_4:Yb,Er/LiYF_4 (green line) and NaGdF_4:Yb,Tm/NaGdF_4:Eu (red line) UCNPs under excitation with 980 nm NIR light.

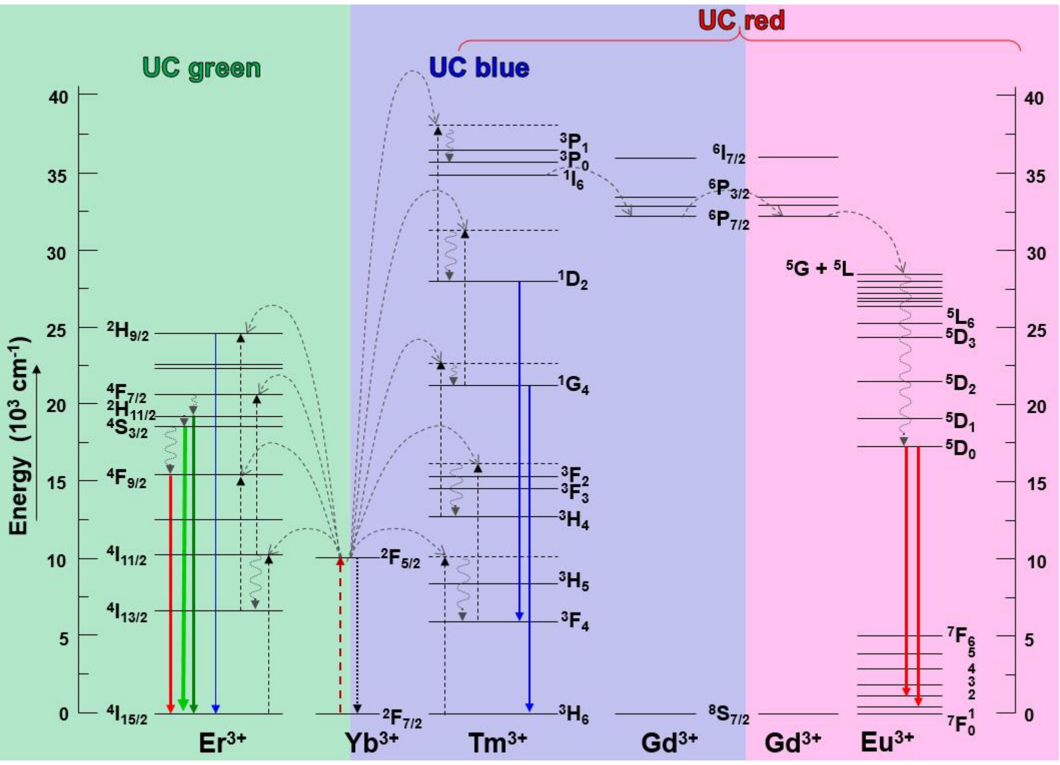

Figure S13. Schematic energy level diagram showing UC green, blue, and red emission from Er^{3+}, Tm^{3+}, and Eu^{3+} ions via energy transfer from Yb^{3+} to Er^{3+}/Tm^{3+} and energy migration through Gd^{3+} ions followed by energy transfer from Gd^{3+} to Eu^{3+}.

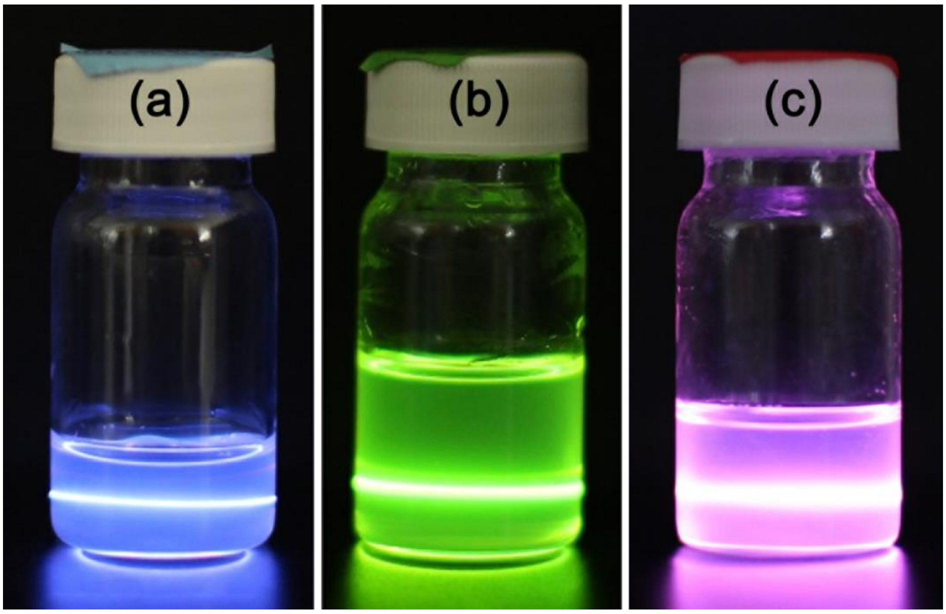

Figure S14. Photographs of core polymer materials mixed with (a) Li(Gd,Y)F_4:Yb,Tm/LiYF_4, (b) Li(Gd,Y)F_4:Yb,Er/LiYF_4 C/S UCNPs, and (c) NaGdF_4:Yb,Tm/NaGdF_4:Eu C/S UCNPs under illumination with 980 nm NIR light.

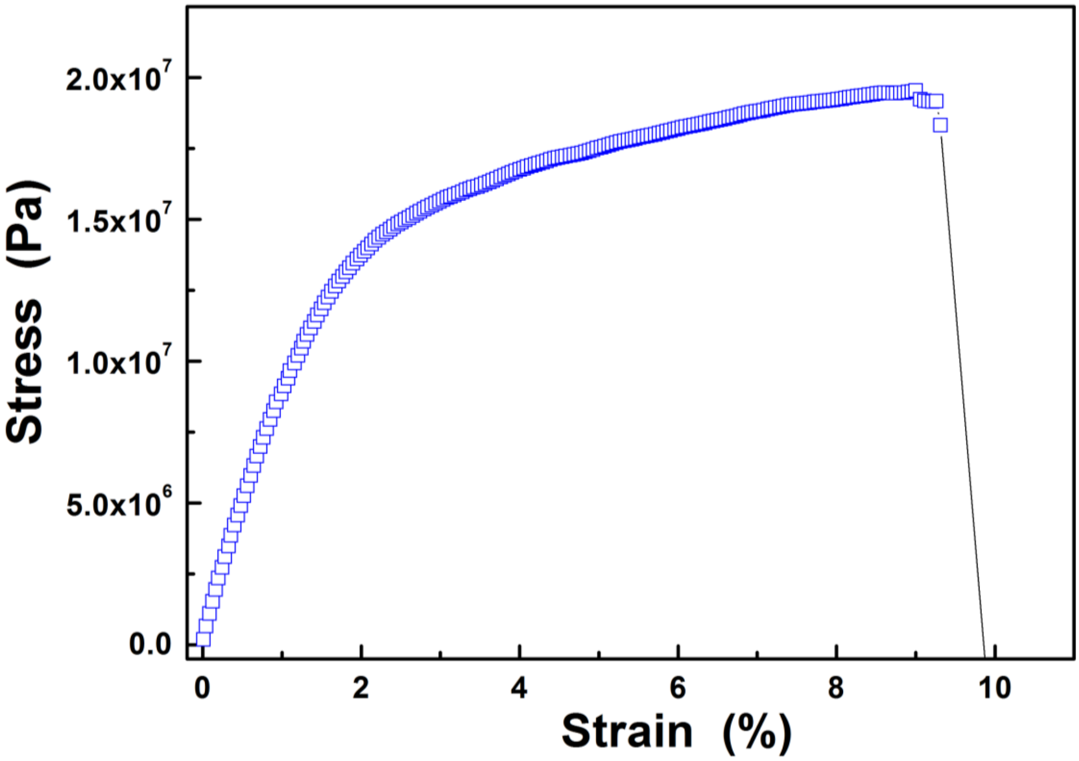

Figure S15. Stress-strain cuve for the polymer substrate. (Mechanical properties of the polymer waveguide were investigated by using a TA Instrument RSA-G2 Solids Analyzer. For the test, rectangular-shaped specimens (thickness: 40 μm) were prepared by using ASTM standard test method for tensile properties of thin polymer sheeting. As the result of a dynamic test under a small oscillatory strain (1%) at a 1 Hz, averaged storage modulus of the specimens was around 424 MPa. The polymer waveguide retains yield strength of 10.7 MPa, ultimate strength of 19.5 MPa, strain at breaking point of 9.3% and elastic limit of ~1.7 %.)

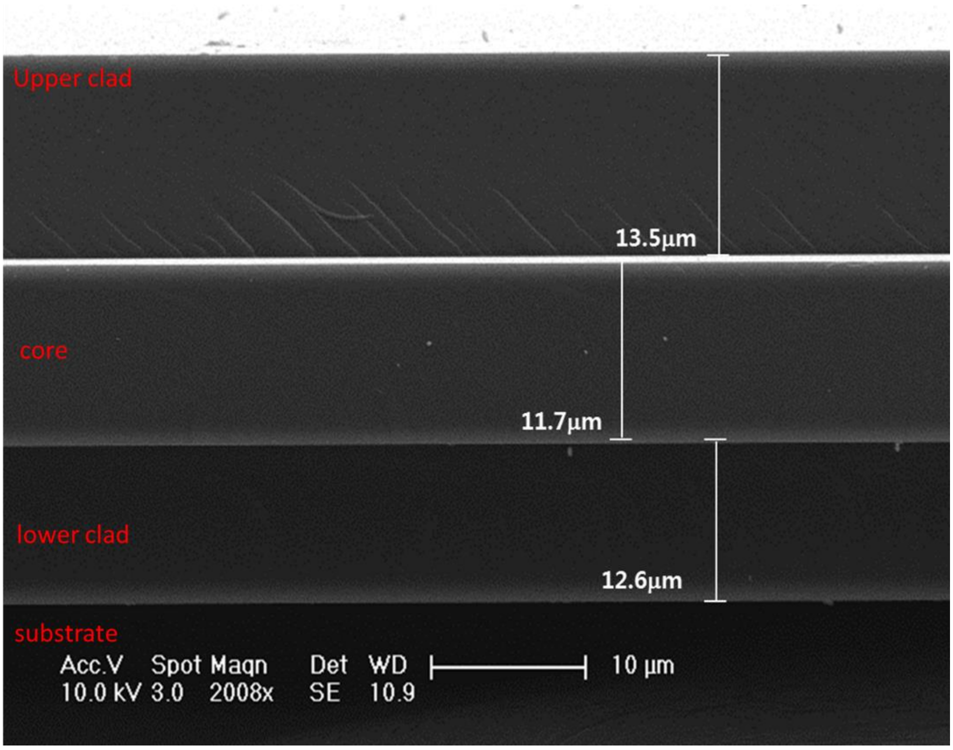

Figure S16. SEM image of cross-section of the stripe-type polymer waveguide

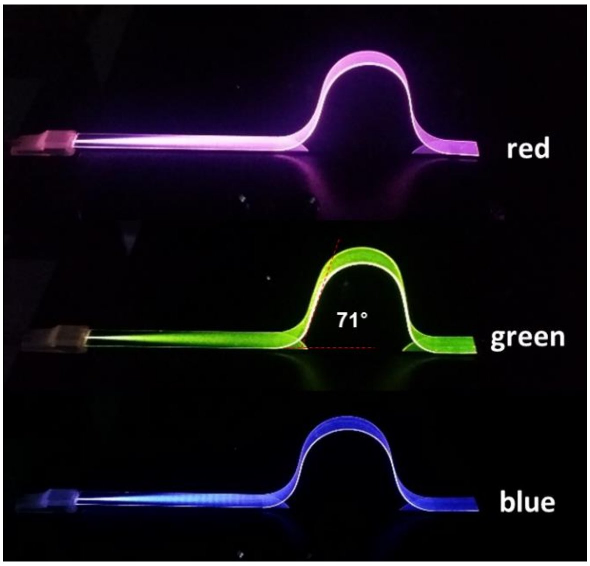

Figure S17. Photographs showing the UCL from largely bended stripe-type polymer waveguides fabricated with blue-emitting Li(Gd,Y)F_4:Yb,Tm/LiYF_4, green-emitting Li(Gd,Y)F_4:Yb,Er/LiYF_4, and red-emitting NaGdF_4:Yb,Tm/NaGdF_4:Eu C/S UCNPs from bottom to top.

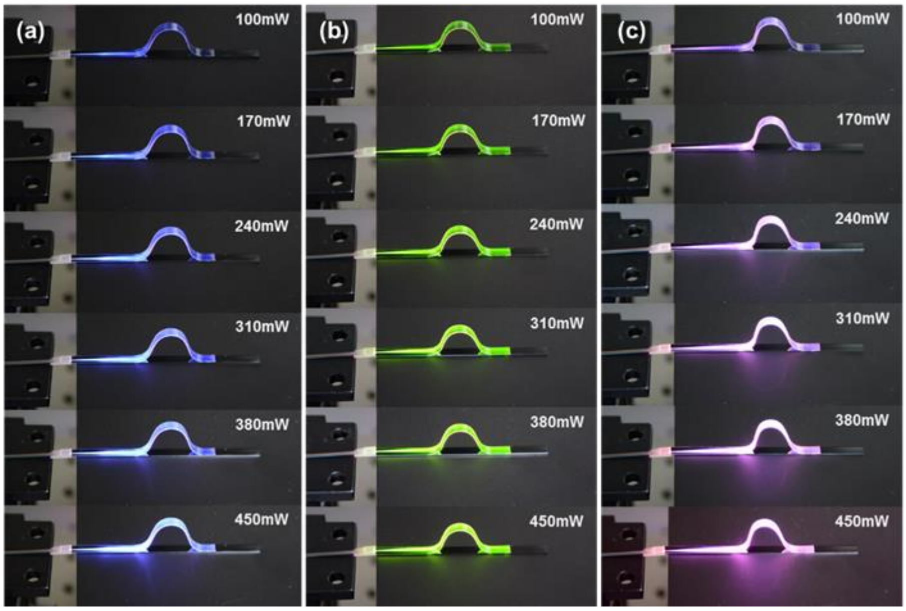

Figure S18. Photographs of stripe-type polymer waveguides fabricated with (a) Li(Gd,Y)F_4:Yb,Tm/LiYF_4, (b) Li(Gd,Y)F_4:Yb,Er/LiYF_4, and (c) NaGdF_4:Yb,Tm/NaGdF_4:Eu C/S UCNPs under coupling with a 980 nm NIR laser with varying incident laser power.

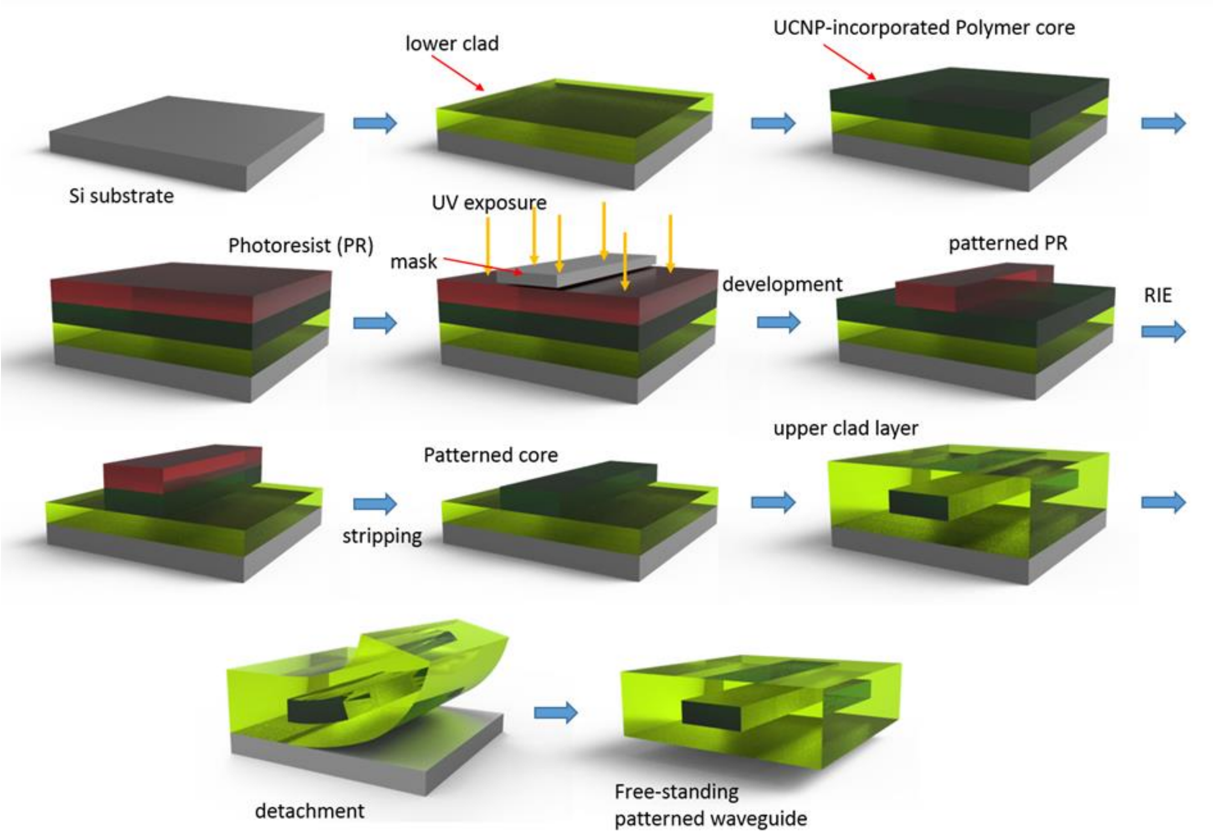

Figure S19. Schematic diagram showing the full procedure of the fabrication of C/S UCNP-incorporated patterned polymer waveguide-based flexible transparent display devices.
